# Protective rules of natural antioxidants against gamma‐induced damage—A review

**DOI:** 10.1002/fsn3.2469

**Published:** 2021-07-21

**Authors:** Qaswaa Y. Jameel, Nameer K. Mohammed

**Affiliations:** ^1^ Department of Food Science Colleges of Agricultural and Forestry Mosul University Mosul Iraq; ^2^ Department of Food Science College of Agriculture Tikrit University Tikrit Iraq

**Keywords:** Food irradiation, free radical, gamma ray, genotoxicity, natural antioxidants, oxidative stress

## Abstract

Phytochemicals accessible in food have demonstrated efficiency against impairment by gamma radiation. The review presented here is an attempt to show the pharmacological outline of the activity of the natural antioxidants and its primary action of molecular mechanism against the damage induced by gamma rays. This research focused on the results of the in vitro dosage of natural antioxidants relationship, and on the correlation of this information with the statistical variables. Moreover, it deliberated the natural compounds which could decrease the unwelcome impacts of gamma radiation and safeguard biological systems from radiation‐stimulated genotoxicity. The outcomes indicated that natural compounds can be utilized as an adjunct to orthodox radiotherapy and cultivate it as an effectual drug for the clinical administration of ailments.

## Graphical abstract

This is the first comprehensive review that collected all naturally occurring antioxidants in foods that act as protective agents against damage caused by exposure to gamma rays. There was no such comprehensive study reviewing the effectiveness of natural antioxidants present in food against the danger of gamma‐ray. This review focused on the antioxidants that act as preventive and therapeutic agents against the danger resulting from radiation.

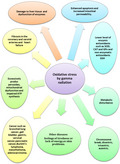

## 1 INTRODUCTION

Gamma rays are the most common structure of therapeutic infrared radiation (IR) (Zeitlin & La Tessa, 2016). A wide gamut of humans and civilizations is subjected to gamma radiations from diverse manmade and natural sources, particularly workforces in the radiation arena and nuclear power sector, patients going through routine radiotherapy, and diagnostic processes along with medical personnel unintentionally subjected to certain level of radiation (Thabet et al., 2020). Gamma radiation stimulates generation of free radicals that affect vitamins activity considerably decreasing the nutritive value of the food damaged with radiation (Hacking & Lane‐Petter, 1968). Irradiation of food containing unsaturated fats produces peroxide which polymerizes and oxidizes the lipids into polymers that are indigestible and which form insoluble plaques that get deposited in the blood vessels (Lubitz et al., 2016). It has been acknowledged that IR could trigger impairment, transformation, alterations in physiological systems, particularly liver tissues (Lu et al., 2009), as well as immune systems (Li et al., 2015). Prolonged contact with Gamma radiation can result in a number of health disorders, especially in the surrounding healthy tissues (Smith et al., 2017), and also increase the permeability of cellular membrane (Zeitlin & La Tessa, 2016).

Earlier research works have indicated that IR can trigger enormous slaying of blood cells like lymphocytes and ceasing of the propagation of hematopoietic progenitors, thus causing overpowering of immune function (Zhao et al., 2014). It can also have a negative impact on antioxidant system resulting in reduced glutathione level and activity of antioxidant enzymes (Zhu et al., 2019), as well as attack intestinal stem cells which results in intestinal epithelium damage, disruption of intestinal barrier function (He et al., 2017), damage of bone‐marrow progenitor cells and gastrointestinal epithelium (Frisby et al., 2007), decrease in small intestine monoamines level—dopamine, epinephrine, norepinephrine, and serotonin (Lu et al., 2016). It is vital to protect the biological systems from lethality or genotoxicity induced by radiation. Herbal as well as natural plant extracts, neutraceuticals, and phytoceuticals have been demonstrated to protect the tissues and cells against the ionizing radiation without unpleasant effects (Harikrishnan et al., 2020). Several research and review articles regarding pharmacological studies have emphasized determining the capacity of natural antioxidants to alleviate micronuclei formation in human (Abdel‐Magied & Elkady, 2019; Srinivasan et al., 2006).

Peripheral blood lymphocytes exposed to gamma‐ray decrease free radical damage produced by ionizing radiation (Smith et al., 2017) and offer protection against gamma‐ray‐induced cellular damage (Ahmadi et al., 2020). However, there is no systematic review to‐date covering antioxidants’ impacts against provoked impairment by gamma radiation. Therefore, this study intends to cover the protective function of the natural antioxidants with emphasis on underlying mechanisms of action which is available in published works regarding the relationship between the dose of natural antioxidant and their effect on oxidative stress by gamma radiation.

## 2 OXIDATIVE STRESS AND DISEASES INDUCED BY GAMMA RADIATION

**FIGURE 1:**
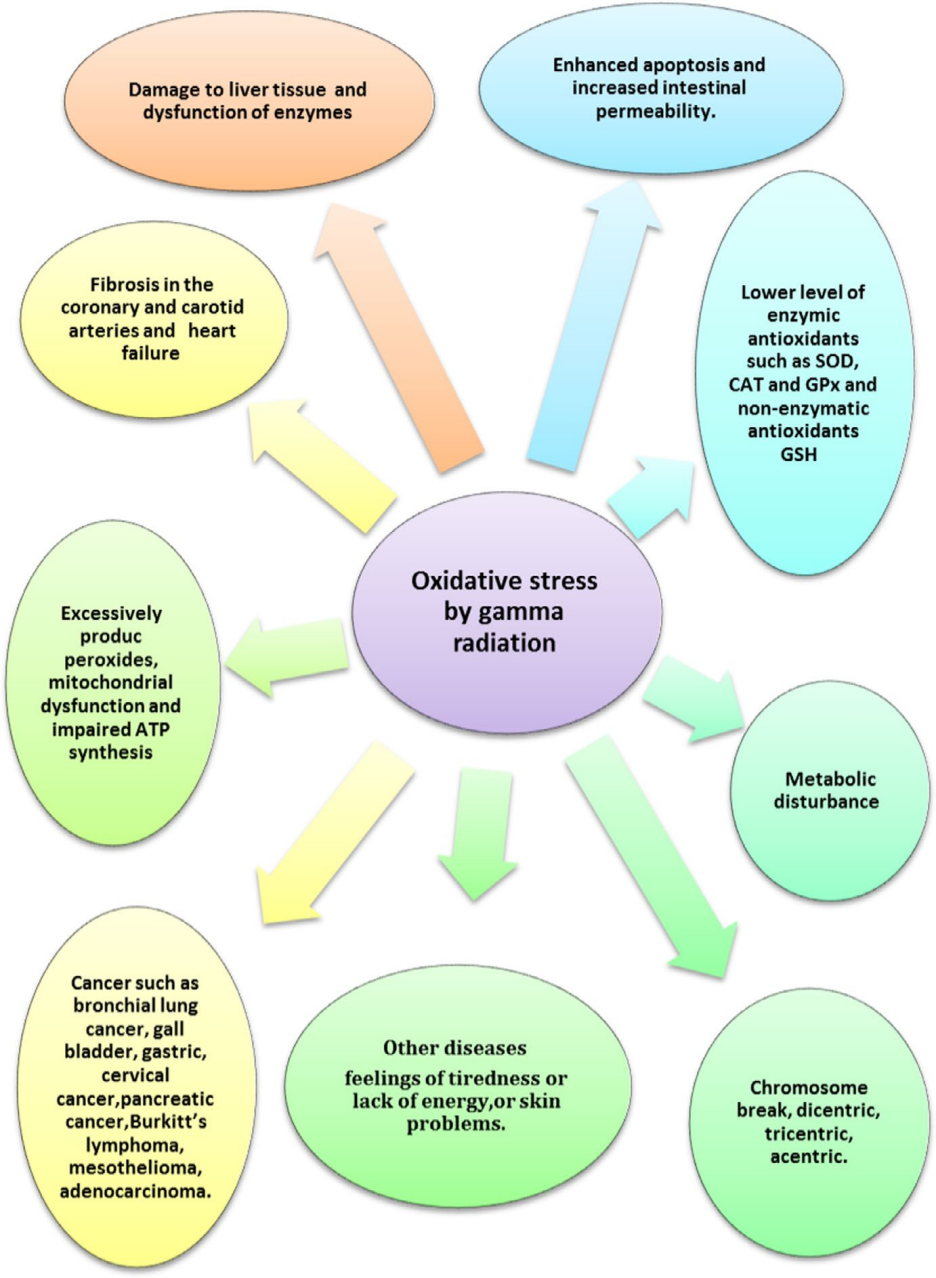
Diverse diseases in different phases of oxidative stress by gamma radiation

Cell apoptosis, mucosal injury, intracellular nucleic acid damage, mitochondrial dysfunction, as well as necrosis, and ulcers are few of the impacts of exposure to Gamma emission (Figure 1). IR exhibits direct as well as indirect interaction with cellular constituents. Direct impacts encompass the direct interface between the DNA and IR. In case of indirect impacts, free radicals like reactive nitrogen species (RNS) and reactive oxygen species (ROS) that are formed from the radiolysis of water molecules intermingle with adjacent DNA molecules to trigger impairments causing disparity of the antioxidant and pro‐oxidant activities, thereby weakening the antioxidant capability of radiation tissues (Yi et al., 2020). Another process through which IR stimulates toxicities is through systemic impacts, also called “nontargeted” impacts. It is a procedure wherein tissues or cells that do not directly intermingle with IR are stimulated with intricate radiation impacts comprising radio adaptive response, bystander impacts, and genomic instability (Najafi et al., 2019). Nonetheless, late impacts such as organ dysfunction, necrosis, death, and cancer might surface months to years after exposure (Peña et al., 2000). Histological observations have demonstrated that accrual of inflammatory cells plays a vital role in the inception of chronic oxidative damage, fibrosis, and inflammation, thus leading to disparities in the normal attributes of the heart as well as a rise in the chances of a heart attack (Taunk et al., 2015). Research has demonstrated that the constant manufacture of free radicals by means of immune system–redox interactions has a key role in the late impacts of radiation exposure in heart tissues, which comprise fibrosis and inflammation (Farhood et al., 2019). Unduly manufactured peroxides, if not removed by endogenous antioxidants, could trigger mitochondrial dysfunction and oxidative stress (Baulch, 2019). Oxidative stress results in disorders that include chronic intestinal damage. Radiation can also increase the oxidative stress in the intestinal epithelial cells (Roy et al., 2016). Raised levels of free oxygen radicals in the mitochondria trigger protein DNA and lipid impairments (El Bakary et al., 2020).

## 3 ROLE OF ANTIOXIDANT AGAINST GAMMA RADIATION

**FIGURE 2:**
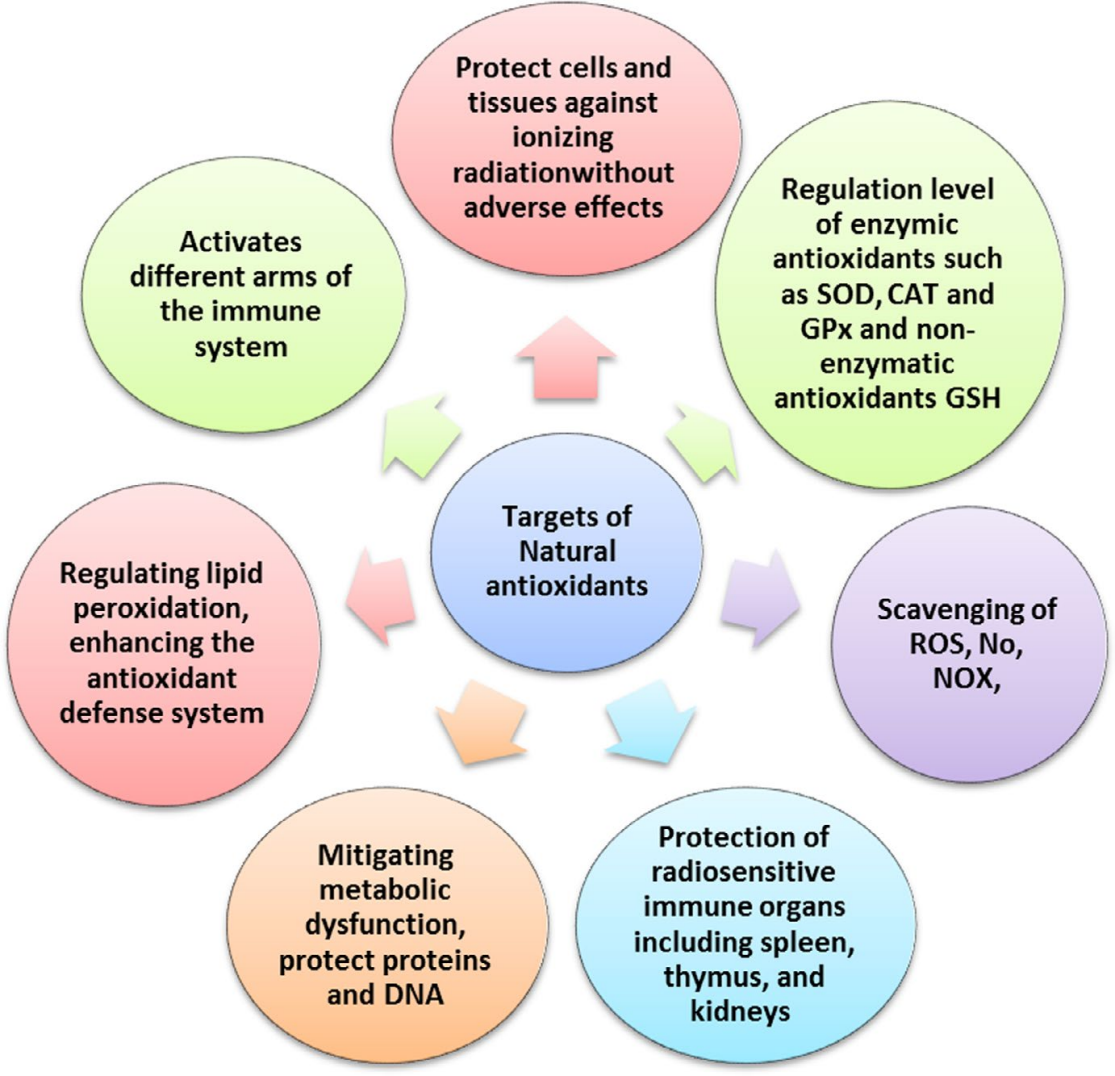
Molecular targets of natural antioxidants and their analogues

Antioxidants are found naturally in several vegetables and fruits (Shaban et al., 2017). Since we are exposed to gamma ray frequently in daily life, it is of utmost importance to develop active and safe radio protectors (Zhu et al., 2019). Phytochemicals observed in plants and foods lacking toxicity have drawn significant attention (Cheng et al., 2019), as against synthetic compounds (Deng et al., 2019). Conversely, as certain antioxidants demonstrate pro‐oxidant activity under specific circumstances, it is more significant to consume natural products comprising different health–functional foodstuffs or antioxidants which are confirmed to be nontoxic, instead of consuming a huge quantity of single antioxidants (Das et al., 2012). Natural antioxidants aid in improving pathological conditions and averting different ailments by assuaging disease advancement (Figure 2). Thus, these kind of plant extracts can be utilized as an aide to traditional radiotherapy (Das et al., 2012). Contact with ionizing radiation provokes the manufacture of reactive oxygen classes, such as hydroxyl radicals, superoxide, singlet oxygen, and hydrogen peroxide. Such free radicals react with vital cellular constituents, like RNA, DNA, membranes, and proteins, causing cell dysfunction and loss of life (Deng et al., 2019). Three endogenous enzymes, which include catalase (CAT), superoxide dismutase (SOD), and glutathione peroxidase (GPx), are accountable for the reactive oxygen species deactivation and are the principal antioxidant system in cells (Sorenson, 2012), and also have the ability to prevent cellular oxidative injury occurring due to presence of free radicals in the body (Choi et al., 2015).

## 4 BIOLOGICAL ACTIVITIES OF NATURAL ANTIOXIDANTS AGAINST DIVERSE CHRONIC DISEASES

In the present research, we review several natural materials that have been examined for defense against oxidative stress induced by gamma radiation. The choice of natural materials is due to their low harmfulness, cost‐effectiveness, and availability. All previous studies are summarized in Table 1 in one glance.

**TABLE 1:** Application of natural antioxidants in different phases of clinical trials

Name of natural product	Dose of gamma ray	Dosage given of antioxidant (bw)	Outcome	In vitro test models	References
Epigallocatechin gallate	6 Gy/min	6.25, 12.5, 25 mg/kg bw	Regulation of the immune system	Male Kunming mice	Yi et al. (2020)
	2, 5 Gy/min	0.183, 0.274, 0.366, 0.549 mg/kg	Reduced cytogenetic damage to bone‐marrow cells and hematological parameters	Male mice	Tiwari et al. (2017)
	3, 5, 7 Gy/s	10, 25, 50 and 100 µm	Protection against radiation‐induced damage	Male Swiss albino mice	Richi et al. (2012)
Gallic acid	4 Gy/min	80 mg/kg	Inhibition of lipid peroxidation	Swiss albino mice	Gandhi & Nair (2005)
	2 Gy/min	50 and 100 μM	Protected the DNA from undergoing the breaks	Human blood	Gandhi & Nair (2005)
Lycopene	6 Gy/min	5 mg/kg/day	Protecting from radiation‐induced oxidative damage	Albino rats	Saada et al. (2010)
	1/2 Gy/min	5 mg/kg	Reduced pathological cellular injuries in the liver	Male albino rats	Fathy Waer & Shalaby (2012)
	4 Gy/min	5 µ/ml	Protection to lymphocytes against radiation‐induced cellular damage	Human blood	Srinivasan et al. (2009)
	10 Gy/min	0.018, 0.015, 0.020 μM	Decreases frequency of chromosomal aberrations	Human blood	Cavusoglu & Yalcin (2009)
Betalain	6 Gy/min	5, 20 and 80 mg/kg	Modulation of the immune system	Male mice	Lu et al. (2009)
Walnut Oligopeptides	8, 3.5 Gy/min	0.22, 0.44 and 0.88 g/kg	Ameliorate acute damage and accelerate its recovery by augment antioxidant defense system	Female mice	Zhu et al. (2019)
Boswellic acid	3 Gy/week	250 mg kg day^−1^	Improvement against damage induced by gamma radiation	Female albino rats	Thabet et al. (2020)
Arabinoxylan rice bran	2 Gy/min	40 mg/kg	Alleviates oxidative stress levels in the serum and radioactive intestinal injury	Male mice	Zhao et al. (2020)
	5 Gy/min	40 mg/kg	Counteract the side effects associated with radiation therapy	Swiss albino mice	Ghoneum et al. (2013)
Fucoidan	5 Gy	20 mg/kg	Prevention and treatment of Gy‐radiation‐induced intestine injury	Male mice	Wu et al. (2020)
Kaempferol	2 Gy/min	5, 15 mg/kg	Inhibiting oxidative stress	Male mice	Wang et al. (2018)
	24 Gy	0.1, 1, 10 µM	Suppress intestinal injury via preventing endothelial apoptosis	Human venous cells	Wang et al. (2018)
Melittin	0.46 Gy/min	500 μg/kg/day	Enhancing the sensitivity of tumor cells to ionizing radiation in addition to protection of normal cells against radiation	Male mice	El Bakary et al. (2020)
Hohenbuehelia serotina	6 Gy/min	50, 100, 200 mg/kg/D	Protection and immunomodulation against radiation‐induced injury	Male mice	Li et al. (2013)
Melatonin	10 Gy/min	100 mg/kg	Preserved villi length and inhibited inflammation	Male Wistar rats	Najafi et al. (2019)
	8 Gy/min	50, 100 mg/kg	Protective effect against radiotherapy‐induced intestinal damage	Male Wistar rats	Musa et al. (2019)
	4 Gy/min	2.5 mg/kg/day	Decreases the oxidative stress and harmful effects on bone‐marrow cells and stimulates the antioxidant enzyme activities	Male albino rats	Assayed & Abd El‐Aty (2009)
Ferulic acid	6.85 kGy/h	50 mg/kg	Abrogates induced oxidative stress and DNA damage	Swiss albino male mice	Das et al. (2017)
	10 Gy	50 mg/kg	Offers protection against gamma radiation‐induced inflammation	Swiss albino mice	Das et al. (2014)
	4 Gy/min	50 mg/kg body weight	Decreasing DNA damage	Male Swiss mice	Maurya & Devasagayam (2013)
	1.66 Gy/min	50 mg/kg body weight	Prevents induced lipid peroxidation	Swiss albino mice	Shanthakumar et al. (2012)
Chrysophyllum cainito	0.64 Gy/min	100 mg/kg day	Attenuates the severity of biochemical disorders of liver	Male albino rats	Sayed et al. (2019)
	0.45 Gy/min	2 g/kg	Decrease the bad side effects of radiotherapy	Male Wistar albino rats	Ulrich and Ziebert (2020)
Salvianolic acid	0.1 Gy/min	5, 12.5, 20 mg/kg	Enhancing immunity and the function of the hematopoietic system	Male & female Kuming mice	Zhou et al. (2019)
Crocin	2 Gy	50 mg/kg bw	Decrease in genotoxicity by gamma radiation	Male Swiss albino mice	Koul & Abraham (2018)

Abbreviations: Gy, gray; min, minute; bw, body weight.

### 4.1 *Effects* *of epigallocatechin gallate against gamma radiation*

Epigallocatechin gallate (EGCG) is the polyphenol present in green tea and has been proved to be an antioxidant (Tiwari et al., 2017). Table 1 provides a summary of the antioxidant effect of EGCG in vitro test models and its ability to reduce the harmful effects caused by gamma radiation by altering histopathological and biochemical biomarkers of oxidative stress. Previous study showed that administering Epigallocatechin gallate in doses of 6.25, 12.5, and 25 mg/kg of body weight 30 days after exposure to radiation with 6 Gy to male Kunming mice improved the transforming capability of the lymphocytes present in the spleen because of the improvement of the impact of immune regulation in vivo, decreased ROS levels, and increased antioxidant enzymes activities including glutathione peroxidase, glutathione, catalase, and superoxide dismutase suppressing peroxidation of lipids, malondialdehyde in the spleen, and liver of irradiated mice (Yi et al., 2020). Another review by (Tiwari et al., 2017) found administering EGCG (0.1833, 0.2749, 0.3666, as well as 0.5499) mg/kg of body weight to male Kunming mice following whole‐body gamma irradiation with 2.5 Gy reduced the DNA damage induced by radiation in bone‐marrow nucleated tissues, prevented HDAC activity, survival compared with the case of whole body lethally irradiated mice, increased acetylation state of histones that triggers the chromatin to achieve a more open confirmation, facilitated DNA repair, and improved recovery rate from hematopoietic depression, particularly in the restoration of lymphocyte percentage.

In another report by (Richi et al., 2012), it was discovered that administering GCG with doses of 10, 25, 50, and 100 µm in order, to Male Swiss albino mice prior to irradiating with doses of (3, 5, 7 Gy) shielded the plasmid DNA, decreased the formation of breaks in DNA strands, decreased the breaks induced in plasmid DNA strands, reduced cell deaths, reduced TBARS level, shielded membrane splenocytes, increased viability of cells, increased SOD and GST enzymes activity, reduced the LDH level, and preserved GSH level.

### 4.2 *Effects* *of gallic acid against gamma radiation*

Gallic acid chemically called 3,4,5‐trihydroxybenzoic acid (Hari Kumar & Kuttan, 2004) is an antioxidant that exists in several fruits and vegetables, and it has various bioactivities such as lipid peroxidation prevention and radical scavenging (Alfei et al., 2020). Various fruits and foodstuffs, including bananas, strawberries, and grapes, comprise different quantities of GA (Kaliora et al., 2013).

Table 1 provides a summary of the antioxidant impact of gallic acid in vitro experimental models and capability to reduce the harmful effects caused by gamma radiation by altering histopathological and biochemical biomarkers of oxidative stress. Review of the literature showed that adding gallic acid with (50, 100 μM) to human blood sample after exposing it to radiation of 2 Gy prevented strand breaks in DNA and partially inhibited the suggesting DNA protection (Gandhi & Nair, 2005). In another report by (Gandhi & Nair, 2005), it was found that administrating Swiss albino mice with 80 mg/kg of gallic acid before exposing them to a dose of 4 Gy for 30 min decreased the lipid peroxidation and cellular DNA damage.

### 4.3 *Effects* *of lycopene against gamma radiation*

Lycopene is an organic pigment produced by microorganisms and plants. It is a carotenoid, which is an acyclic β‐carotene–isomer, and does not have vitamin A activity (Kelloff et al., 1999). It is a greatly unsaturated, straight chain hydrocarbon having conjugated and 2 nonconjugated double bonds (Müller et al., 2011). It is present in several vegetables and fruits such as papaya, pink grapefruit, pink guava, and water melon (Müller et al., 2011). Nonetheless, processed tomato products and tomatoes are a major lycopene source in diet (Stahl et al., 2006). Recent attraction of lycopene has concentrated on its antioxidant characteristics (Stahl et al., 2006), and it has found that adding lycopene to chemotherapy and radiotherapy in patients leads to a positive outcome (Tsen et al., 2006). A number of studies have confirmed that lycopene possesses antioxidant properties against cell membrane damage induced by exposure to gamma radiation (Wood et al., 2008), and it is also antitumor (Huang et al., 2008). Exposure of animals to ionizing radiations causes development of a dose‐dependent, complex series of changes, which include reduction in villus height, number of villi, reduction in crypt cells amount, as well as damage of goblet and epithelial cells (Akpolat et al., 2009). Table 1 provides a summary of the lycopene antioxidant effects in vitro experimental models and capability to reduce the harmful effects caused by gamma radiation by altering histopathological and biochemical biomarkers of oxidative stress.

The review of literature has demonstrated that administering lycopene orally in dose of (5 mg/kg BW) to male albino rats, through gavages, for 7 consecutive days, prior to exposure of gamma radiation to whole body with 6 Gy reduced the activity of XO and the TBARS level with noteworthy simultaneous increase in the CAT and SOD enzyme activity, and reduced the GSH content, compared to their corresponding amounts in irradiated rats. Furthermore, a substantial increase was recorded in the monoamines level related to a significant decrease in the MAO activity. As a result, lycopene would safeguard cellular membrane from lipid peroxidation induced by radiation, thus maintaining the structure of small intestine and inhibiting monoamines release (Saada et al., 2010). Fathy Waer & Shalaby (2012) report demonstrated lycopene effects with dosage of 5 mg/kg daily on adult and healthy male albino rats exposed to radiation of (1/2) Gy. It showed that Lycopene induces great improvement in the cellular organoids structure against cellular damaged induced by the gamma radiation. The cytoplasm sustained its granulation and gained its usual pattern with revival of ribosomes. The nucleotic mitochondria also gets normal. Other remarkable finding in this research was that droplets of lipid still found in Lycopene‐treated mammals in considerable averages in comparison to γ‐irradiated rats. This is probably because of the antioxidant effect of Lycopene in reducing lipid peroxidation and enhancing antioxidant status that prevents cell damage.

Another review by Srinivasan et al. (2009) demonstrated lycopene effect at a dose of (5 g/ml) on samples of human blood that were sterilely collected in heparinized sterilized glass tubes from median cubital vein of healthy nonsmokers of 22–25 years, with (4 Gy). Preprocessing of lycopene with gamma‐irradiated lymphocytes caused reduction in lipid peroxidation, increased CAT, GPx, and SOD activities in lymphocytes. Lycopene modulates phase II enzymes such as glutathione reductase (GR), glutathione S‐transferase (GST) and glutathione peroxidase (GPx), and levels of GSH preventing radiation damage to lymphocytes by inhibiting lipid peroxidation and formation of breaks in DNA strands induced by free radicals.

Another review by Cavusoglu and Yalcin (Cavusoglu & Yalcin, 2009) demonstrated lycopene effect for doses (0.018, 0.015, and 0.020 μM) on human lymphocytes. Samples of peripheral blood were gathered from ten healthy individuals, and they were selected randomly from a bunch of nonsmokers. Among these individuals were 5 males and 5 females, and the individuals’ average age was (20–25) years. These samples were treated with 10 Gy irradiation for a duration of 30 min. The lymphocytes were treated with lycopene doses which caused a substantial decrease in chromosomal aberrations frequency. Addition of Lycopene caused beneficial outcomes against genotoxic injury induced by gamma radiation in lymphocytes. Moreover, it was suggested that lycopene can avert carcinogenesis by safeguarding vital molecules like DNA. It was also showed that addition of lycopene increases myocardial infarction, which is regarded as a sensitive indicator of antioxidant activity of lycopene.

### 4.4 *Effects* *of betalains against gamma radiation*

Betalains are regarded as pigments that are water‐soluble and can be categorized as betacyanins (Gandhi & Nair, 2005). A rich source pertaining to betalain pigments is red beet. As a natural additive, betalain pigment can be employed for cosmetics and food (Janiszewska, 2014). These pigments are categorized as antioxidants, that is, compounds that can prevent or delay the processes of oxidation, subsequently preventing diseases like cancer as well as cardiovascular diseases (Ravichandran et al., 2013). Betalains contain hydroxyl groups, which enable increase in scavenging activity of free radicals (Gandía‐Herrero et al., 2009). When rats were fed with betalain‐rich red beet juice, it minimized the action of the hepatotoxic compounds N‐nitrosodiethylamine, 7,12‐dimethylbenz (a) anthracene and carbon tetrachloride, because of the enhanced antioxidant status as well as increased expression pertaining to quinine reductase (Szaefer et al., 2014). Table 1 provides a summary of the antioxidant effect pertaining to Betalains in vitro test models as well as the capability to minimize the harmful impact due to gamma radiation by changing histopathological and biochemical biomarkers with regards to oxidative stress.

Previous study, the oral administration of betalains to male mice at doses of 5, 20 and 80 mg/kg, for 30 consecutive days prior to whole‐body gamma irradiation with 6 Gy, as well as once daily for 3 consecutive days postirradiation, resulted in decrease of white blood cells, as well as increase in the micronucleus rate pertaining to polychromatic erythrocytes. The micronucleus rate pertaining to polychromatic erythrocytes in the mice’ bone marrow treated with 5, 20, or 80 mg/kg of betalains extracted from red beets was found to suppress SOD as well as GSHPx activities in the spleen, liver, and kidney. Administration of betalains orally, which were extracted from red beets, was found to restore the SOD activity partially in three different tissues in a dose‐dependent manner, suppress the lipid oxidation that was induced via irradiation, restore the catalase activity in all three organs, as well as reduce the level of MDA to almost that of the control group (Lu et al., 2009).

### 4.5 *Effects* *of walnut oligopeptides against gamma radiation*

Walnut as a crop is grown worldwide and contains different functional components possessing excellent antioxidant activity (Liu et al., 2019), which also offer protection against cardiovascular diseases (Sánchez‐González et al., 2017). In addition, walnuts have been found to prevent liver fat accumulation (Choi et al., 2016). The walnut extract also demonstrated to confer protection with regards to the cholinergic system and antioxidant system by keeping a check on superoxide dismutase contents, malondialdehyde levels, decreased glutathione contents, acetylcholinesterase activity, and acetylcholine levels (Kim et al., 2020). In a rat model, walnuts have also demonstrated to confer protection via anti‐inflammatory action against ethanol‐induced gastric mucosal injury (Liu et al., 2020). Table 1 provides a summary of the antioxidants effect pertaining to walnut oligopeptides as well as the capability to minimize the harmful impact of gamma radiation by changing histopathological and biochemical biomarkers pertaining to oxidative stress in in vitro test models. In previous study, the oral administration of walnut oligopeptides to female mice at doses of 0.22, 0.44, and 0.88 g/kg BW via drinking water for 14 consecutive days before and after dose of whole‐body irradiation with 8, 3.5 Gy was found to accelerated hematopoietic recovery, augment the antioxidant defense system as well as demonstrate a considerable trend toward achieving higher survival rate and less weight loss versus nonadministrated control mice. Also, administration of walnut oligopeptides was found to limit the inflammatory cascade and IR‐induced splenocyte apoptosis, promote epithelial integrity, and decrease intestine epithelial barrier dysfunction. This advocates that administration of Walnut Oligopeptides is key to limiting IR‐induced inflammatory cascade by minimizing the DNA double‐strand breaks, promoting epithelial integrity when there is acute tissue damage due to IR and minimizing intestine epithelial barrier dysfunction, all of which aid in preventing the action of inflammation (Zhu et al., 2019).

### 4.6 *Effects* *of boswellic acids against gamma radiation*

Boswellic acid can be defined as a phytochemical (Deng et al., 2019), which includes a series of pentacyclic triterpene molecules. It can be isolated from the gum resin of Boswellia carteri and Boswellia serrata produced by the trees called as Indian olibanum (Roy et al., 2016). Boswellic acids are potent, novel, specific anti‐inflammatory agents, which can be attributed to the nonredox inhibition pertaining to 5‐lipoxygenase enzyme (Iram et al., 2017). As a traditional remedy, it has wide application pertaining to different ailments, particularly inflammatory diseases, asthma (Roy et al., 2016), treatment of colds, coughs, bronchitis, hoarseness, and dyspnea (Vuddanda et al., 2016). Table 1 provides a summary of the antioxidant effect pertaining to Boswellic acids in in vitro test models as well as the capability to minimize the harmful effects of gamma radiation by changing histopathological and biochemical biomarkers pertaining to oxidative stress. As per literature study, oral administration of boswellic acid to female albino rats at dose levels of 250 mg kg^−1^ day^−1^ for 4 weeks with 3 Gy/week (dose of 0.662 Gy/min during experiment) demonstrated considerable improvement against damage caused by IR as confirmed by histopathological examination and biochemical investigations of liver and heart based on alleviation of hepatic oxidative stress markers and hepatotoxicity. In irradiated rats, this was found to help prevent cardiac oxidative stress as well as enhance cardiotoxicity biomarkers through a distinct decline in hepatic glutathione (GSH) and malondialdehyde (MDA) levels, and maintenance of serum total glycerol, aspartate aminotransferase (AST), and alanine aminotransferase (ALT) levels in animals (Thabet et al., 2020).

### 4.7 *Effects* *of arabinoxylan rice bran (MGN‐3/Biobran) on gamma radiation*

MGN‐3/Biobran can be defined as a natural product extracted from rice bran hemicelluloses (Badr El‐Din et al., 2020), a polysaccharide that includes beta‐1,4‐xylosyl hemicellulose. The key chemical structure pertaining to MGN‐3 includes an arabinoxylan possessing a xylose in its main chain and in its side chain with an arabinose polymer (Ooi et al., 2018). Table 1 provides a summary of the antioxidant effect pertaining to arabinoxylan rice bran (MGN‐3/Biobran) in in vitro test models as well as the capability to reduce the harmful impacts of gamma radiation by changing histopathological and biochemical biomarkers pertaining to oxidative stress. In the literature, oral administration of arabinoxylan rice bran (MGN‐3/Biobran) to mice at dose levels of 40 mg/kg body weight before irradiation with 2 Gy/min for 5 min as well as a single dose of 10 Gy has demonstrated to provide protection to all areas of the intestine against radiation damage, helped restore the intestinal permeability to reach normal physiological levels, and considerably decreased malondialdehyde (MDA), reactive oxygen species (ROS), and hydrogen peroxide (H_2_O_2_) levels in serum, colon, and jejunum. However, antioxidant indicators like glutathione peroxidase, catalase, superoxide dismutase, as well as total antioxidant capacity activities have been found to increase significantly in the serum, jejunal, and colonic mucosa simultaneously. Furthermore, MGN‐3 has been found to effectively attenuate the radiation‐induced alterations pertaining to the intestinal epithelial mitochondrial function, inflammatory response, oxidative stress, intestinal permeability, and apoptosis in mice (Zhao et al., 2020).

In a different report by (Ghoneum et al., 2013), administration of 40 mg/kg of MGN‐3 for 4 weeks to Swiss albino mice, which would then receive gamma radiation of 5 Gy, avoids immune dysfunction related to irradiation, that is, MGN‐3 behaves as both an immune modulator and a radioprotector, confers protection against γ‐irradiation‐induced reduction pertaining to hemoglobin values, improves the blood cellular radio resistance, protects against γ‐irradiation, keeps a check on the white blood cells (WBC) count as well as overall maintenance of hematopoietic tissues, safeguards against radiation‐induced increase of MDA content, and offers protection against irradiation‐induced loss of organ and body weight.

### 4.8 *Effects* *of fucoidan against gamma radiation*

In many Asian countries, Fucoidan is regarded as a cost‐effective food supplement. It can be isolated from seaweed or marine brown macroalgae (Wang et al., 2019) and contains water‐soluble, anionic heteropolysaccharide (van Weelden et al., 2019). Fucoidan's chemical composition with regards to various types of brown algae is different. Apart from sulfate and fucose, it also includes other monosaccharides (galactose, mannose, glucose, etc.) as well as glucuronic acid (van Weelden et al., 2019). Various bioactivities are exhibited by oligofucoidan, like immunomodulatory, gastric protective effects, antithrombotic, and anti‐inflammatory activities (Dos et al., 2015). It helps avoid lipid peroxidation as well as provides protection against oxidative damage of the cellular membrane (Wu et al., 2014), offers antiviral properties, improves immune functions, antithrombotic (Li et al., 2020), and decreases damage done to human leukemia cells (Nita & Grzybowski, 2016).

Table 1 provides a summary of the antioxidants effect pertaining to fucoidan in vitro test models as well as the capability to reduce the harmful impacts caused by gamma radiation by changing histopathological and biochemical biomarkers pertaining to oxidative stress. Previous study showed that administration of oligofucoidan at a dose level of 20 mg/kg body weight orally post 24 hr and prior to irradiation with gamma 5 Gy radiation to mice for 10 consecutive day demonstrated averting induced lipid peroxidation as well as helped restore intestinal enzymatic antioxidants with regards to CAT, SOD, and GPx, nonenzymatic antioxidants with regards to GSH activities in tissues samples, alleviate the loss of crypt and villi within the small intestine and minimize radiation‐induced death in mice (Wu et al., 2020).

### 4.9 Effects of kaempferol against gamma radiation

A low molecular weight flavonoid is Kaempferol (3,5,7‐trihydroxy‐2‐ (4‐hydroxyphenyl)‐4‐H‐1‐benzopyran‐4‐one), which includes a phenolic ring and hydroxyl group that are prone to donating an electron or a hydrogen atom in order to free radicals, and thus extending the conjugate aromatic system pertaining to delocalization of an unpaired electron (Dai & Mumper, 2010). Kaempferol has extensively existed in several types of medicinal herbs, fruits, and vegetables (Wang et al., 2015), like cabbage, broccoli, tomatoes, beans, strawberries, grapes, and tea (Calderón‐Montaño et al., 2011), Amburana cearensis, aloe vera, and Cassia angustigolia (Gouveia et al., 2016). Kaempferol is said to possess anti‐inflammatory, antioxidant, and immunomodulatory characteristics (Martino et al., 2014), like inhibiting human monoamine oxidases‐A pertaining to the peripheral tissues and brain as well as inhibiting cell growth of pancreatic cancer (Lee & Kim, 2016) and (Gidaro et al., 2016). Table 1 provides a summary of the antioxidants effect pertaining to Kaempferol for in vitro test models and the capability to reduce the harmful impacts of gamma radiation by changing histopathological and biochemical biomarkers pertaining to oxidative stress.

Previous study demonstrated that oral gavage administration of kaempferol to male mice at a dose levels of 5 and 15 mg/kg body weight for 30 days before irradiation with gamma (7 and 8.5 Gy) radiation can significantly mitigate the loss of weight post radiation, as well as attenuate radiation‐induced injury with regards to spleen, jejunal, and thymus. Pretreatment with kaempferol may also considerably inhibit radiation‐induced cell apoptosis in mice models (Wang et al., 2018). As per a separate report by (Wang et al., 2018), pretreatment performed for 24 hr on human umbilical venous endothelial cells by (10 µm) kaempferol post 24 Gy gamma radiation was found to decrease apoptosis and increase human umbilical venous endothelial cells. It helped deal with oxidative stress as well as restore the protein level pertaining to human umbilical venous endothelial cells postradiation in order to confer protection against radiation‐induced injury via scavenging of free radicals in tissues and cells. This also promoted reduction in the level of ROS, NO, and MDA, decreased radiation‐induced tissue injury and apoptosis and increased the level of SOD.

### 4.10 Effects of melittin against gamma radiation

Bee venom chiefly includes melittin component, which can be defined as a peptide of 26 amino acids (Hwang, Kim and Bae, 2015). It is also regarded as a key active ingredient found in honeybee venom (Chen et al., 2016). Melittin includes many pharmacological, biological and toxicological activities, including antifungal and antibacterial activities, as well as possible antitumor activity. With regards to cancer treatment, radiotherapy is regarded as a key modality (Son et al., 2007). Melittin possesses antitumor properties and is also regarded as a natural therapeutics for cancer chemotherapy (Jeong et al., 2014). Melittin has potential anticancer impacts against breast, ovarian (Jo et al., 2012), leukemia, hepatocellular carcinoma (Hoshina & Marin‐Morales, 2014), and melanoma, prostate (Liu et al., 2013).

Table 1 provides a summary or the antioxidants effect pertaining to melittin for in vitro test models as well as the capability to reduce the harmful impact caused due to gamma radiation by changing histopathological and biochemical biomarkers pertaining to oxidative stress. According to previous study, it is observed that the administration of Melittin in a dose of (500 μg/kg body weight/day) injected intraperitoneally to mice with 1 cm^3^ solid tumor of Ehrlich ascites carcinoma for 21 days, while the mice is undergoing irradiation with gamma (at a dose rate of 0.46 Gy/min), then Melittin in honeybee venom has the potential to inhibit lipid peroxidation and exhibit a substantial hydroxyl radical scavenging activity, along with obstruction of tumor growth, augmentation of tumor cells apoptosis, and specific enhancements in the state of tumor cells penetration and infiltration. A considerable decline in the level of apoptotic molecule (caspase‐3) is also observed along with development in apoptotic regulators (caspase‐3 activity) and free radicals content, and normal cells redox tone catalase (El Bakary et al., 2020).

### 4.11 Effects of polysaccharide of hohenbuehelia serotina against gamma radiation

Hohenbuehelia serotina refers to a kind of mushroom that grows in the North Hemisphere. In traditional China, this mushroom is consumed as a medicinal fungus (Xie et al., 2016). A key component of the Hohenbuehelia serotina is polysaccharide, which is proven to have significant antitumor and antioxidant benefits (Li et al., 2014). Polysaccharides are long chain molecules, composed of ketoses or aldoses, and bonded with glycosidic links (Kanagasabapathy et al., 2014). As concluded in various research works, the bioactivities of polysaccharides were strongly driven by their structural characteristics. The polysaccharides with the main chain of (1→3)‐β‐glucan could be regarded as immunomodulator, while the polysaccharides with the backbone of (1→3)‐α‐glucan generally behaved as tumor inhibitor (Singdevsachan et al., 2016). The structural characteristics of the polysaccharides predominantly included the monosaccharide composition, molecular weight, types of glycoside bond, extent of polymerization, and degree of branching, as well as the three‐dimensional advanced conformation, and all these traits determined the biological activities of the polysaccharides (Jing et al., 2015). Polysaccharide of Hohenbuehelia serotina had substantial biological activities, like immunomodulatory (Wong et al., 2011), antioxidant (Wu et al., 2013), antitumor (Li et al., 2012), and lowering blood (Zhang et al., 2013).

Table 1 summarizes the antioxidant behavior of the polysaccharide of Hohenbuehelia serotina in in vitro test models and its capability to diminish the harmful impact of gamma radiation by altering the histopathological and biochemical biomarkers of oxidative stress. As observed in the literature study, the administration of polysaccharide of Hohenbuehelia Serotina through oral gavage in a dose of (50, 100, and 200) mg/kg of body weight for 14 days before exposing the male mice to gamma (6 Gy) radiation resulted in an increase in the level of catalase and superoxide dismutase in kidney and liver, reduction in the level of malondialdehyde, and enhancement in the immunomodulatory activities that involve protection of the number of WBCs, proliferation of splenocytes, and protection of splenocytes against the damage caused by radiation (Li et al., 2013).

### 4.12 *Effects* *of melatonin against gamma radiation*

Melatonin (N‐acetyl‐5‐methoxytryptamine) is generally present in cereals, walnuts, tomatoes, legumes, ginger, olive, and pineapple (Meng et al., 2017). Melatonin has the ability to minimize the DNA damage responses and apoptosis with its antioxidant and anti‐inflammatory effects, which eventually results in reduction of cell death and consequent inflammation (Shabeeb et al., 2018). Table 1 presents the antioxidant effect of Melatonin in in vitro test models and its capability to reduce the damaging impacts of gamma radiation by changing the histopathological and biochemical biomarkers of oxidative stress. According to the literature study, the administration of melatonin through oral gavage of male Wistar rats in a dose of 100 mg/kg in 24 hr and 30 min before exposing them to 10 Gy gamma rays and continued for 3 days after irradiation protects ileum against radiation toxicity, protects vessels and goblet cells, prevents infiltration of macrophages and lymphocytes or mucositis, stimulates antioxidant defense in cells, protects against radiation‐induced apoptosis, reduces inhibitory effect on radiation‐induced apoptosis, preserves villi length, and inhibits inflammation in the intestine (Najafi et al., 2019).

In another report (Musa et al., 2019), it was observed that administration of 50, 100 mg/kg of melatonin to abdominal regions of male Wistar rats for 30 min before exposing them to 8 Gy gamma rays from gamma radiation resulted in reduction of levels of malondialdehyde (MDA), protection of the tissues against radiotherapy‐induced intestinal damage, prevention of enteritis, and improvement of catalase and superoxide dismutase activities of the tissues. In a different report by (Assayed & Abd El‐Aty, 2009), it was observed that administration of 2.5 mg/kg of melatonin to male albino rats intraperitoneally daily for 5 consecutive days before exposing them to 4 Gy from gamma radiation led to protection of the chromosomes and bone‐marrow cells of those rats, and activation of certain cellular enzymatic antioxidant defense mechanisms, which involve glutathione peroxidase, catalase, and superoxide dismutase, and amelioration of the radiation‐induced enhancement of 4‐hydroxyalkenal plus malondialdehyde and protein carbonyls, along with protection of the genome against DNA damage.

### 4.13 Effects of ferulic acid against gamma radiation

Ferulic acid (FA) can be defined as a phytochemical that is found in fruits and vegetables like sweet corn, tomatoes, rice bran, wheat, and broccoli (Srinivasan, Sudheer, et al., 2006). It includes a strong antioxidant that enables nitric oxide scavenging activity as well as hydroxyl radical scavenging activity. It has become a popular choice due to its properties such as cost‐effectiveness (Ou & Kwok, 2004). Table 1 provides a summary of the antioxidant effect pertaining to ferulic acid in vitro test models as well as the ability to minimize the harmful effects of gamma radiation by adjusting the histopathological and biochemical biomarkers pertaining to oxidative stress. A previous study showed that administration of ferulic acid at a dose of 50 mg/kg body weight orally once daily, prior to exposing a single dose of 10 Gy whole‐body irradiation in swiss albino mice, was found to considerably prevent the radiation‐mediated damage pertaining to plasmid DNA, activate nonhomologous end‐joining DNA (repair process) as well as significantly increase the antioxidant enzymes via scavenging reactive oxygen species (ROS) (Das et al., 2017).

As per a report by (Das et al., 2014), administration of 50 mg/kg body weight of ferulic acid to Swiss albino mice for 5 days before exposure to a single dose of 10 Gy was found to increase the activity pertaining to superoxide dismutase, glutathione and catalase levels, decrease the lipid peroxidation, mitigate the radiation induced via inflammatory response, prohibit the rise in cycloxygenase‐2 (Cox‐2) protein, induce lipid peroxidation in liver, and increase the level of proinflammatory cytokines interleukin‐6 (IL‐6) and tumor necrosis factor‐alpha (TNF‐a) in serum.

In a different report by (Maurya & Devasagayam, 2013), intraperitoneal administration of 50 mg/kg body weight of ferulic acid to Male Swiss mice before and immediately post whole‐body Ɣ‐irradiation of mice with 4 Gy was found to decrease the level of DNA strand breaks in murine peripheral blood leukocytes and improve the survival of mice, which could be due to the minimization of DNA damage and micronuclei formation. In a report by (Shanthakumar et al., 2012), intraperitoneal administration of 50 mg/kg body weight of ferulic acid to Male Swiss mice post whole‐body Ɣ‐irradiation of mice with 8 Gy, for five consecutive days was found to increase the levels of catalase, super oxide dismutase and glutathione peroxidase activities, decrease the depletion level of glutathione concentration in liver, spleen and intestine, and decrease the number of inflammatory, dead and mitotic cells and preserved villus height.

### 4.14 Effects of chrysophyllum cainito extract against gamma radiation

The fruits of Chrysophyllum cainito L., related to the family Sapotaceae (Wang et al., 1997), have health benefits and their attributes comprise antioxidant (Fernandez‐Panchon et al., 2008), antidiabetic, and antihypertensive properties (Van et al., 2018), and anticancer (Jing et al., 2015). Table 1 provides a summary of the antioxidant effect pertaining to *Chrysophyllum cainito* extract with in vitro test models as well as the capability to reduce the harmful impact of gamma radiation by modifying the histopathological and biochemical biomarkers pertaining to oxidative stress. Another study showed that administration of *Chrysophyllum cainito* extract at a dose of 100 mg/kg body weight orally prior to and post gamma irradiation with 6 Gy to male albino rats showed amelioration pertaining to liver enzymes activities (ALT, AST, and ALP) as well as glucose level, reduction of MDA level, enhancement in kidney function, liver function and lipid profile, enhancement in selenium level pertaining to the liver, and reduction in triglyceride and cholesterol levels (Sayed et al., 2019).

### 4.15 Effects of salvianolic acid B against gamma radiation

Salvia miltiorrhiza is the source from which salvianolic acid is extracted (Rahmani et al., 2020). Traditional Chinese medicine uses Salvia miltiorrhiza and is among the extensively used herbs (Shen et al., 2017). Salvianolic acid is an effective antioxidant that captures free radicals of oxygen (Qian et al., 2014). Contemporary pharmacological research indicates that Salvia protects vascular endothelial cells, has anti‑arrhythmic properties, enhances blood flow to the coronary artery, removes free radicals, enhances microcirculation, slows atherosclerosis, protects against pulmonary fibrosis and lipid peroxidation, and protects liver cells (Paik et al., 2011). Table 1 lists the antioxidant properties of Salvianolic acid B specific to in vitro tests and the ability to the chemical to reduce the adverse effects of gamma radiation by changing histopathological and biochemical indicators of oxidative stress. The literature indicates that 14‐day intraperitoneal administration of Salvianolic acid at 5, 12.5, and 20 mg per kg body weight after exposure to 6 Gy (0.1 Gy/min, for 185 s) to male and female Kuming mice offers protection against damage to protein structure, decreases ROS levels, restores SOD activity, and reduces apoptosis. Furthermore, MDA levels are decreased, while peripheral RBC and WBC levels, thymus, and spleen indices are enhanced.

### 4.16 *Effects* *of crocin against gamma radiation*

Crocin is a water‐soluble pigment carotenoid that is extracted from the stigma of the Crocus sativus L. (Saffron) flower (Ben Salem et al., 2016). It protects against DNA damage (Rahaiee et al., 2015), and possesses antihyperlipidemic (Tamaddonfard et al., 2013) and antidepressant characteristics (Rahaiee et al., 2015). Crocin is found to have cancer‐opposing activity against specific tumor cells (Bakshi et al., 2017). It reduces the damage caused by DNA breakage caused by hydroxyl radicals produced due to radiation (Kudryasheva & Rozhko, 2015). There is documentation indicating the protective effects of crocin against radiation poisoning of the kidneys, heart, liver, and skeletal muscles (Samarghandian et al., 2016). Table 1 lists the antioxidant effect of Crocin specific to in vitro tests. It decreases the adverse effects of gamma radiation by changing the histopathological and biochemical markers of oxidative stress. The literature indicates that oral Crocin administration in male Swiss albino mice at 50 mg per kg body weight in the 2 days following 2 Gy whole‐body radiation exposure leads to enhanced free radical removal. Moreover, micro‐nucleated polychromatic erythrocytes and genotoxicity decrease; there is lesser damage to testicular tissue. Crocin provides protection DNA strand breakage caused by hydroxyl free radicals (Koul & Abraham, 2018).

## 5 CONCLUSION

Gamma radiation exposure is risky; it should be reduced to decrease the risks. The present study focused on the use of natural antioxidants that are considered to have therapeutic effects against radiation. The emphasis is on substances that are cost‐effective and readily available. Individuals working around radiation sources or in the radiotherapy field should be provided natural antioxidants. Empirical data indicate that the reviewed substances offer protection against gamma radiation. The use of gamma radiation and chemotherapeutic drugs like Doxorubiein for treating cancer causes significant damage to the liver. Orally administered natural antioxidants can prevent or reduce the harm caused by such drugs or radiation therapy. These observations indicate the efficacy of supplementing natural antioxidants for protection against the effects of gamma radiation. The need for other medicines can be reduced, and quality of life can be enhanced. The study showed that the natural substances did not lead to toxicity; hence, potential clinical trial safety concerns are addressed. Nevertheless, the effectiveness of such substances remains to be assessed during clinical trials. We propose that these substances be assessed in future clinical trials to establish their efficacy and practicality in clinical settings. Moreover, clinical trials are expected to yield more information concerning experimental outcomes on human cells. Furthermore, the mechanisms through which radiation protection is achieved need to be clarified. The study results might lead to a noteworthy increase in the therapeutic index and the effectiveness of radiotherapy. We assessed the correlation between natural antioxidants and their effects on oxidative stress caused by gamma rays. The data are correlated with statistical factors. It is shown that natural substances may be used to augment radiotherapy. Further studies are necessary to investigate the therapeutic effects of the natural antioxidants on gamma‐induced damage as a nutraceutical supplement.

## CONFLICT OF INTEREST

The authors declare that they do not have any conflict of interest.

## AUTHOR CONTRIBUTIONS

**Qaswaa Jameel:** Data curation (equal); Formal analysis (equal); Investigation (equal); Methodology (equal); Project administration (equal); Writing‐original draft (lead). **Nameer Mohammed1:** Formal analysis (equal); Methodology (equal); Project administration (equal); Supervision (lead); Writing‐review & editing (equal).

## ETHICAL APPROVAL

The study did not involve any human or animal testing.

## Data Availability

All authors consent that raw data presented in this review are available after publication.
